# Efficacy of a theory-based and tailored mHealth intervention promoting walking behavior: a preliminary randomized controlled trial

**DOI:** 10.1038/s41598-025-09634-3

**Published:** 2025-07-18

**Authors:** Roberta Adorni, Francesco Zanatta, Silvia Serino, Maria Elide Vanutelli, Daniela Caso, Marco D’Addario, Patrizia Steca

**Affiliations:** 1 Department of Psychology, University of Milano-Bicocca, Milan, Italy; 2 Department of Humanities, University of Naples Federico II, Naples, Italy

**Keywords:** Walking behavior, Health communication, Well-being, Behavior change, mHealth, Psychology, Risk factors

## Abstract

Physical inactivity is one of the core risk factors for non-communicable diseases. Scaling up health interventions using theoretically-driven and smart-enabled approaches is critical to strengthening prevention strategies. This study evaluated the efficacy of an ad-hoc developed mobile health (mHealth) intervention, which adopts a theory-based and tailored communication approach and targets walking behavior change. A convenience sample of physically inactive adults (n = 193) participated in a 30-day randomized trial. Before the intervention, psychological variables selected from the Health Action Process Approach (HAPA) were evaluated to profile participants and develop tailored materials. Allocation (1:1:1) was performed to one of three conditions: (i) tailored communication based on the HAPA model (HAPA-T group); (ii) non-tailored communication focused on wellbeing (Wellbeing-NT group); (iii) no-communication group. All participants were also exposed to goal-setting (i.e., reaching 7,000 steps daily) and self-monitoring. A repeated measures ANOVA was performed to monitor the mean weekly steps over the 30-days trial period within and among the three conditions. Overall, participants reported significant improvements in walking behavior over the trial period. Although no significant outcome differences were observed among the three experimental conditions, differential patterns of walking emerged from the study groups, with the HAPA-T group showing a wider increase at the final time-point. The intervention showed a significant overall impact, unveiling crucial procedural strengths and limitations. These were discussed to orient optimized implementation for future digital full-scale trials.

*Trial Protocol Registration* ClinicalTrials.gov registration ID NCT05620888 (17^th^ November 2022; https://clinicaltrials.gov/study/NCT05620888).

## Introduction

The high prevalence of chronic non-communicable diseases (NCDs) represents one of the main health challenges of today’s society. NCDs cause approximately 74% of the world’s annual deaths. Among these, cardiovascular diseases represent the leading cause of mortality globally^1^. The onset of these conditions is multifactorial, and the main risk factors are modifiable. Therefore, the most effective way to prevent NCDs is to counteract the main modifiable risk factors by promoting healthy lifestyles^2^.

Increasing physical activity is critical to reducing the burden of NCDs. The World Health Organization (WHO) recommends at least 150 min of moderate to vigorous-intensity physical activity each week^3^. In addition to increasing physical activity, it is essential to limit the time spent sitting by alternating it, for example, with light-intensity walking breaks^4^. Evidence suggests that excessive and prolonged sitting can lead to diverse negative consequences for health, including insulin resistance, vascular dysfunction, reduced cardiorespiratory capacity, the shift of muscle fiber from oxidative to glycolytic type, deposition of mass and visceral fat, blood lipid concentrations, and inflammation^5^. Current evidence indicates that 7,000 daily steps, which corresponds to approximately 60 min of walking, are sufficient to benefit health, reducing sensibly all-cause mortality^6–8^.

The connection between a physically inactive lifestyle and poor health outcomes is well-established and widely recognized^2,5^. However, it remains a significant concern that 27% of adults worldwide do not meet the recommended levels of physical activity^9^. Therefore, the development and implementation of effective interventions to increase physical activity in line with the Global Action Plan on Physical Activity 2018–2030^10^ represents an urgent challenge.

With the widespread use of technology, digital-based trials, particularly mobile health (mHealth) interventions, have become increasingly popular for promoting physical activity. This is due to their cost-effectiveness and broad reach^11,12^. mHealth trials focused on behavior change have been widely conducted, with reports showing promising but preliminary evidence regarding the effectiveness of digital tools^13–15^. A little-considered aspect is their capacity to deliver compelling and persuasive content that can result in significant and enduring behavioral change^16–20^. In this context, insights from non-digital literature, such as tailored health communication and theoretical models predicting behavioral change, can be valuable.

Tailored health communication involves using a combination of communication strategies to encourage behavioral change in a specific individual by considering that individual’s unique characteristics^21^. Tailored messages can help change behavior by providing personally relevant information and feedback^22,23^.

In a previous study^24^, our research team showed that using printed tailored communication and a theory-based approach effectively increased physical activity over 12 months in a group of patients with arterial hypertension. The patients who received tailored printed material showed a gradual increase in physical activity over twelve months. On the other hand, patients who received non-tailored printed material exhibited an initial increase in physical activity levels after six months of intervention, followed by a decrease. The tailoring, described in detail in a previous article^25^, was based on the variables of the Health Action Process Approach (HAPA)^26^, which is one of the most influential behavioral change models. HAPA defines the implementation and maintenance of health behaviors as the result of a stage process. Each stage implies the role of specific socio-cognitive variables, which can facilitate or hinder the entire process. During the “motivational” phase, individuals develop an intention to change, which is explained by the confidence in their ability to change (i.e., action self-efficacy), their perception of risk related to the current behavior (i.e., risk perception), and their beliefs about the positive and negative outcomes associated with the behavior to be changed (i.e., outcome expectancies). Once the intention is developed, individuals enter the “volitional” phase, during which the behavior materializes and is maintained. This phase is determined by planning strategies, confidence in one’s ability to maintain the newly adopted behavior (i.e., maintenance self-efficacy), and confidence to return to implementing the healthy behavior following an interruption (i.e., recovery self-efficacy). The validity of this model in predicting physical activity change has been demonstrated^27–29^.

A recent meta-analysis^30^ examined the effectiveness of tailored mHealth interventions for promoting physical activity in adults. Out of the sixteen studies, ten reported significant improvements in physical activity levels for intervention groups compared to controls. The most common tailoring method involved behavioral feedback and setting personal preferences, like the timing of messages. Only three studies based their tailoring on theoretical premises: one reported higher physical activity levels in intervention groups, while the other two found no between-group differences.

As already mentioned, in our previous study^24^, we obtained encouraging results by combining printed-tailored communication^21–23^ with a theoretical approach validated in the literature^26–29^. In numerous prior mHealth studies, interventions were not tailored based on theoretical constructs^30^. Therefore, our current study aimed to scale-up our approach by evaluating the efficacy of a theoretically-driven and tailored communication intervention through the preliminary implementation of an ad-hoc developed mobile application ("*MyPocketHealth*"). Consistently with our previous study, walking behavior change was targeted as primary study outcome.

Research has shown that while persuasive communication can lead to a change in attitude and subsequently influence behavioral change, its impact is more significant when combined with prompts to use specific goal-setting (setting a clear goal, such as "taking 7,000 steps a day") and self-monitoring strategies^12,31^. Accordingly, we also aimed to test the implementation of a mobile application that can provide users with a structured and satisfying experience. As a result, we also referred to the Behavior Change Techniques (BCTs) Taxonomy to design our intervention^32^, and we selected those that showed to be effective in promoting physical activity, both in non-digital^33,34^ and digital contexts^35,36^, namely goal setting and behavioral self-monitoring.

In summary, the primary aims of this study were to:

i. evaluate the efficacy of a theory-based and tailored communication intervention delivered through an ad-hoc mobile application
ii. explore the specific effects of tailoring on behavioral change (i.e., pursue the goal of 7000 steps a day), comparing our innovative tailored communication technique centered on psychological predictors of behavioral change according to HAPA^24,25^ to a generic non-tailored communication technique focused on the wellbeing associated with walking, as tested in previous studies^35,38^

To this end, three experimental groups were compared:

i. Goal setting + self-monitoring + tailored communication using the Health Action Process Approach (HAPA-T group)
ii. Goal setting + self-monitoring + non-tailored communication focusing on wellbeing (Wellbeing-NT group)
iii. Goal setting + self-monitoring without any additional communication (no-communication group)

Because we compared two communication protocols that have shown their effectiveness in different contexts, we did not make a specific prediction about which type of communication was more persuasive. However, we hypothesized that participants in the first two experimental conditions would report improvement in walking compared to the participants in the no-communication group.

A secondary aim of the study was to explore the associations between the HAPA model variables and the walking behavior, providing additional insights into the social-cognitive factors underlying the trajectories estimated^27–29^.

## Methods

### Participants

A convenience sampling method was applied to invite participants from the social network of the researchers and their students. Participants were invited through word of mouth and by distributing flyers at the university. They were eligible according to the following criteria: age between 18 and 70 years, good physical health, physically inactive lifestyle, level of education sufficient to understand the procedure, and having a smartphone (either iPhone or Android). Participants were excluded if taking at least 7,000 steps a day, or having a sustained physical activity (as measured by IPAQ, see the section “Assessment of Walking Behavior”), or having a particular pharmacological treatment or disease that prevented them from voluntarily changing their lifestyle without an indication from the treating physician.

The sample size was calculated a priori by resorting to power analysis^39^ through G*Power Software Version 3.1.9.7^40^. The required sample size was calculated to perform a repeated measures ANOVA with within-between interaction and the following parameters: f = 0.25 (medium effect size), α = 0.05, power = 0.95; number of groups = 3; number of measurements = 4. The sample size calculated was 171 individuals.

The study was approved by the local commission for minimal-risk studies of the Department of Psychology of the University of Milano-Bicocca (RM-2021–482), and it was pre-registered at the ClinicalTrials.gov Registry (Trial ID: NCT05620888). All participants were informed about the study purposes and procedures and voluntarily provided the consent to participate. The present research was conducted in accordance with the Declaration of Helsinki and following CONSORT guidelines and all relevant regulations.

### Design and procedure

**Fig. 1:**
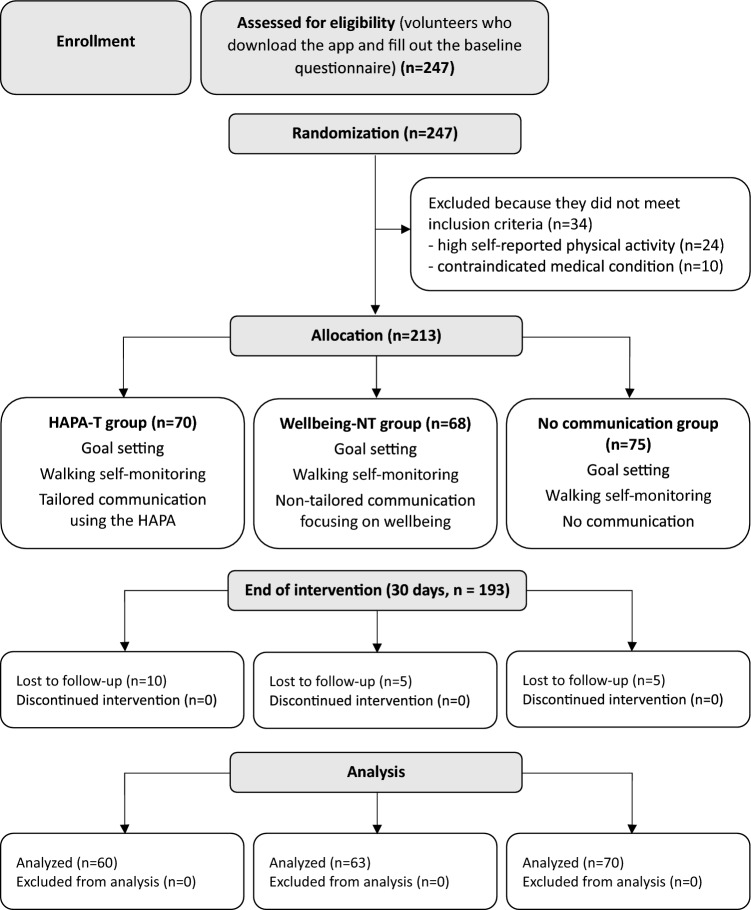
Flowchart of the study.

The study has a prospective, three-arm, parallel-group, randomized design. It included a baseline assessment and a 30-days trial period (Fig. 1).

We designed a mobile application ("*MyPocketHealth*") that invited users to participate in a physical activity program requiring them to reach a daily goal of 7,000 steps and to self-monitor their behavior by entering the number of steps taken each day into the app. Communication was handled by sending one notification per day through the app. The app notification tool strategy was selected to increase the chance of better engaging participants, as suggested by recent evidence on the use of behavior promoting applications^37^. The app was developed by iMoobyte S.R.L., an innovative software company based in Italy, and tested in version 1.0 .It was built for both iOS and Android operating systems through Flutter framework (v.3.24.3) to support push notification systems across platforms. Additionally, a web-based Content Management System (CMS) was developed using React framework and hosted on Firebase, providing a centralized and scalable solution for content updates and application management.

Each volunteer was asked to download the app and join the study. The app automatically assigned each volunteer to one of the three experimental conditions via a round-robin single-blinded randomization (1:1:1) and provided them with a link to complete an online questionnaire administered through the Qualtrics platform. The questionnaire’s landing page explained the purposes and procedures of the study, in addition to obtaining informed consent from participants. Initial questions assessed the eligibility of participants. Afterward, eligible participants completed queries to collect sociodemographic and psychological information according to the HAPA model^26,28^.

Participants assigned to the "HAPA-T group" received a daily tailored notification. Tailoring was designed using the HAPA^26,28^. The underlying idea was that individuals go through different phases on their path to behavioral change. Therefore, interventions may be more efficient if tailored to these phases. Specifically, the content was tailored to the following variables: intention to achieve the 7,000 steps per day, positive outcome expectancies, health risk perception, self-efficacy, action planning, and coping planning. The order of sending notifications was consistent with the sequential order of the HAPA model. The contents of the notifications were written by three authors of this study (P.S., M.D., and F.Z.), all experts in health psychology. The notifications provided feedback about the participants’ position in the HAPA. Each variable in the model corresponded to one or more notifications and included suggestions to motivate change. A low score corresponds to a score between 1 and 3 for variables measured on a 5-point Likert scale and between 1 and 4 for variables measured on a 7-point Likert scale. For instance, if participants reported high negative outcome expectancies in the baseline questionnaire, they would receive a notification highlighting that they could achieve more than expected by walking regularly (i.e., "It can certainly be challenging to start walking regularly, but you will see that it will help you feel fitter over time!"). The Supplementary Material (See Table S1) illustrates the tailoring scheme and some examples of notifications.

Participants assigned to the "Wellbeing-NT group" received a daily non-tailored notification on wellbeing from taking at least 7,000 steps daily (See Table S2 of the Supplementary Material). An example item is: “Walking regularly every day helps you reduce bad mood.” HAPA-T and Wellbeing-NT notifications were identical in structure, number of words, and graphic format. Only the degree of customization of the information content varied: generic for the Wellbeing-NT group and tailored for the HAPA-T group.

"No-communication group" received no notifications throughout the trial period.

**Fig. 2:**
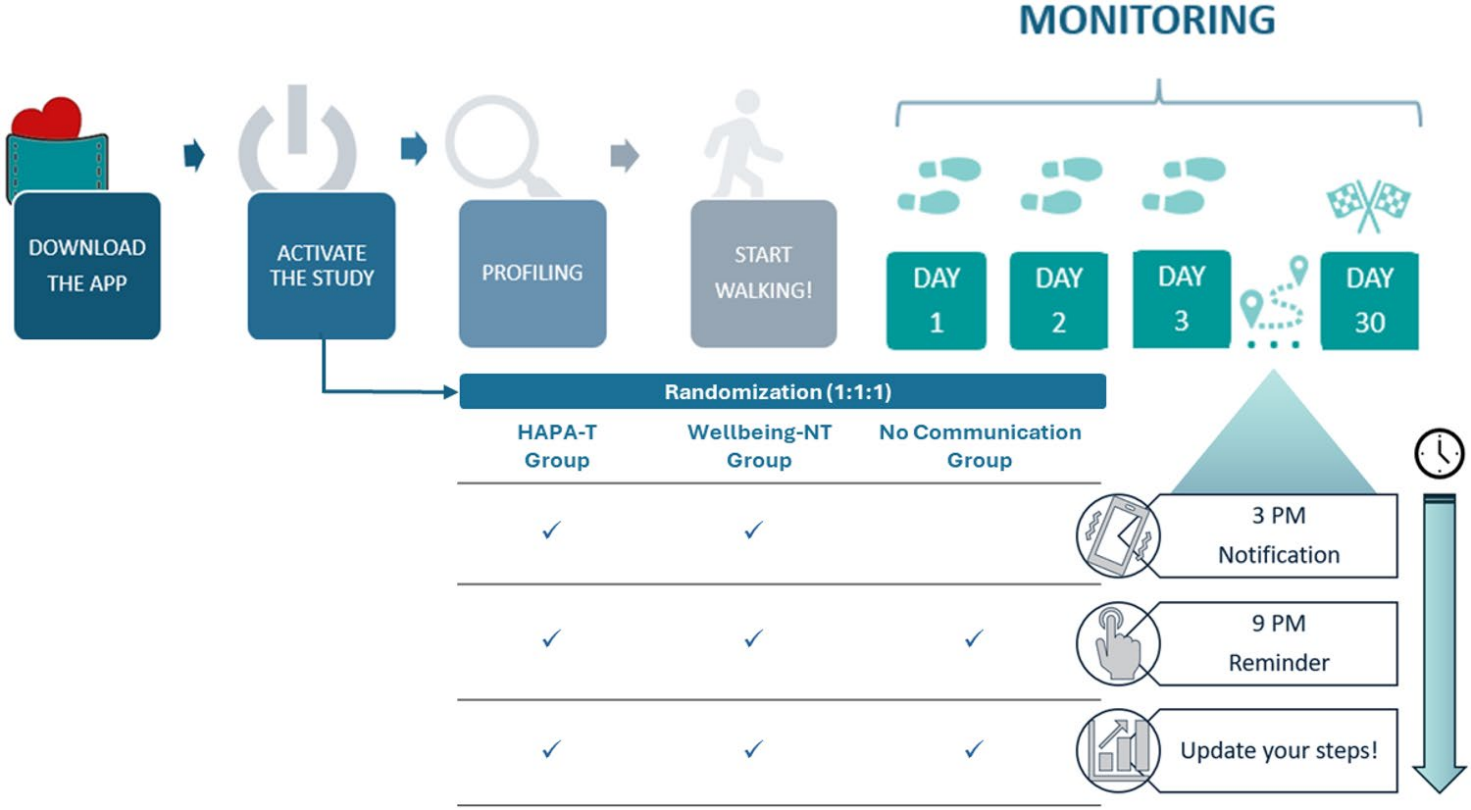
Trial procedure.

All participants, independently of the experimental condition, received a daily reminder to enter the number of steps taken during the day in a dedicated app section (i.e., walking self-monitoring). The steps gradually populated a graph depicting the temporal trend of one’s behavior and the set objective. Figure 2 illustrates the procedure.

### Assessment of walking behavior

The International Physical Activity Questionnaire (IPAQ)^41,42^ was administered in the initial online questionnaire to identify inactive volunteers. The IPAQ is a 7-item scale that investigates three specific types of physical activity (walking, moderate-intensity activity, vigorous-intensity activity) and sedentary activity in terms of frequency (days per week) and duration (time per day). The items are structured to provide separate scores for each type of activity and a combined total score, which describes the overall activity level in terms of median MET-minutes/week. The score is obtained by calculating the amount of physical activity weighted by the energy requirement defined in METs (i.e., multiples of resting metabolism). For the present study, a cut-off of 3,000 MET-minutes/week was set, which identifies respondents classified as inactive or minimally-active (see www.ipaq.ki.se). Volunteers exceeding this threshold were excluded from participation.

Walking behavior was self-monitored daily. Every evening, the participants received an app reminder and entered the number of steps taken in a dedicated section of the app, based on the data reported on the smartwatch or a native smartphone app. The number of steps registered on the first day was considered as baseline value of the study outcome. The mean number of steps taken in 10-day time intervals was then calculated (from the second to the tenth day, from the eleventh to the twentieth day, and from the twenty-first to the thirtieth day). The four measurements (day 1, days 2 to 10, days 11 to 20, and days 21 to 30) were compared to verify whether an increase in mean daily steps could be observed over time.

### Health action process approach (HAPA) model variables

The questionnaire administered online at baseline assessed the following variables, taken from the HAPA model^26^ and adapted from previous studies^28,35^. The list of HAPA model constructs, with an example question and an example of tailored notification for each construct can be found in the Supplementary Material.

Action self-efficacy: Participants indicated confidence in their abilities to walk regularly through a single item on a 5-point Likert scale, where 1 = not capable and 5 = fully capable. The higher the score, the higher the action self-efficacy.

Positive and negative outcome expectancies: Participants indicated their positive (3 items) and negative (3 items) expectancies about the health, emotional, and social effects of taking at least 7,000 steps a day on a 7-point Likert scale, where 1 = in no way, 7 = very much. The score was calculated as the mean of the three items’ scores. The higher the score, the higher the expectancies. The scales demonstrated a sufficient internal consistency (McDonald’s ω = 0.634 and ω = 0.659 respectively).

Health risk perception: Participants indicated how exposed they feel to health risks (six items) concerning their unhealthy behavior (i.e., how little they walk) on a 7-point Likert scale, where 1 = in no way, 7 = very much. The score was calculated as the mean of the six items’ scores. The higher the score, the higher the health risk perception. The scale demonstrated an adequate internal consistency (McDonald’s ω = 0.918).

Intention to change walking behavior: Participants indicated how much they intended to walk regularly (take at least 7,000 steps per day at a moderate speed) through a single item on a 7-point Likert scale, where 1 = totally disagree, 7 = totally agree. The higher the score, the higher the intention to walk regularly.

Planning: Participants indicated if they had a detailed plan concerning six concrete aspects of achieving the goal of 7,000 steps per day (for example, when and where to walk) on a 7-point Likert scale, where 1 = in no way, 7 = very much. The score was calculated as the mean of the six items’ scores. The higher the score, the higher the planning. The scale demonstrated an adequate internal consistency (McDonald’s ω = 0.823).

Maintenance self-efficacy: Participants indicated confidence in their ability to maintain the new healthier behavior despite obstacles and difficulties through ten items on a 7-point Likert scale, where 1 = in no way, 7 = very much. The score was calculated as the mean of the ten items’ scores. The higher the score, the higher the maintenance self-efficacy. The scale demonstrated an adequate internal consistency (McDonald’s ω = 0.850).

Recovery self-efficacy: Participants indicate confidence in their ability to regain healthy behavior if a lapse occurs through a single item on a 5-point Likert scale, where 1 = not capable and 5 = fully capable. The higher the score, the higher the maintenance self-efficacy.

### Data analysis

Descriptive statistics were calculated on the sample’s sociodemographic, psychological, and behavioral characteristics. Mean and standard deviation (SD) were reported for continuous variables, and percentages were reported for categorical variables. The normal distribution of the data was tested by calculating skewness and kurtosis indices; the recommended range of ± 2 and ± 7 was considered for normality, respectively^43^. McDonald’s ω was calculated to estimate the internal consistency of the psychological scales^44,45^. All statistical tests were two-tailed, and a p-value of ≤ 0.05 was considered statistically significant. Attrition is expected in longitudinal studies^46^. Accordingly, Mann–Whitney U and Chi-square tests were used to identify possible dissimilarities between the participants who concluded the study and the volunteers who dropped out. Moreover, it was verified whether randomization was successful, testing for possible dissimilarities between the participants allocated in the three experimental groups. A correlation analysis was then performed to explore the association of sociodemographic characteristics (i.e., age and gender) and the mean number of steps. A correlation coefficient lower than |0.2| was considered negligible, whereas a coefficient higher than |0.2| (suggestive of a weak correlation) represented a sufficient reason to control the following analysis for the effect of that characteristic, or in other words, to include the specific characteristic as a further between-subjects factor in the ANOVA. A repeated measures ANOVA test was finally performed to monitor the mean weekly steps over the 30-days trial period and directly compare the behavioral variations detected among the three experimental groups. A 4 × 3 two-way mixed factorial design was used, with a within-subject factor (the four-time intervals) and a between-subject factor (the three experimental groups). The main and interaction effects of time and experimental groups were analyzed. Partial eta squared (η^2^_p_) was used as a measure of effect size and Bonferroni post-hoc tests were used to note any statistically significant differences in the mean weekly step number across time and experimental groups.

In addition, a Pearson’s correlation matrix was generated to explore the associations between the HAPA model variables and the walking behavior over time. We identified correlations as effect size measures according to Cohen’s guidelines^39^. Correlations were interpreted as weak (|.10|< r <|.29|), moderate (|.30|< r <|.49|), or strong (|.50|< r <|1|). All analyses were performed using Jamovi (Version 2.2.5, The Jamovi project, 2021, retrieved from https://www.jamovi.org) and IBM SPSS Statistics, version 29 (SPSS, Chicago, IL, USA).

## Results

### Preliminarily analyses and description of the study sample

247 volunteers downloaded the application (June 2023–July 2024) and were assessed for eligibility. 34 were excluded because they did not meet the inclusion criteria: 24 volunteers reported high physical activity, and 10 reported a contraindicated medical condition. The remaining 213 volunteers were included in the study (Fig. 1). Of these, 20 volunteers (9.4%) completed the baseline assessment but dropped out during the 30 days of step monitoring, or in other words, they stopped entering steps into the app.

Volunteers who dropped out of the study did not differ significantly from the participants in their sociodemographic characteristics, namely age and gender, nor the experimental group allocation. They did not differ in their self-reported physical activity and the mean number of steps declared on the first intervention day. Volunteers who dropped out of the study did not differ significantly from the participants in the psychological variables considered in the following analyses, except their intention to change walking behavior (U = 1362; p < 0.05; rank biserial correlation = 0.29; achieved power = 0.70). Volunteers who dropped out declared less intention to walk regularly (mean = 4.30; SD = 2.11) than participants who completed the study (mean = 5.38; SD = 1.43).

The final sample consisted of 193 participants, with 125 women (65%), and they had a mean age of 34 years (range 19–70, SD: 14.2). Most participants earned a high school diploma (41%) and lived with their families, i.e., with their parents (42%) or their spouse and children (28%). The complete description of the participants’ sociodemographic characteristics is reported in Table 1. The mean total score of the IPAQ was 952 MET-minutes/week (range 0–2970, SD: 719).

**Table 1:** Sociodemographic characteristics of the sample (n = 193).

Sociodemographic Variables	
Age (mean ± SD; range)	34.3 ± 14.2 (19–70)
Gender	
Male	68 (35.2)
Female	125 (64.8)
Education, n(%)	
Middle school diploma	12 (6.2)
High school diploma	79 (40.9)
Bachelor’s degree	55 (28.5)
Master’s degree	36 (18.7)
Postgraduate training	11 (5.7)
Living condition, n(%)	
Alone	11 (5.7)
With parents	80 (41.5)
With room mates	21 (10.9)
With the spouse or partner	25 (13.0)
With the spouse or partner and children	53 (27.5)
With the children	3 (1.6)

Means and standard deviations (SD) are reported for age. Frequencies (n) and percentages (%) are reported for the other variables.

Participants allocated in the three experimental conditions did not differ significantly in terms of age and gender, nor in their self-reported physical activity and the mean number of steps declared on the first intervention day. Moreover, no significant differences were found in the psychological variables.

### Walking behavior change

The results of the preliminary correlation analysis (Table 2) evidenced no association between the sociodemographic variables, i.e., age and gender, and walking behavior over time. Therefore, sociodemographic variables were not included in the analysis described below.

**Table 2:** Correlations between socio-demographic characteristics of the sample (n = 193) and walking behavior.

Walking behavior	Age (Pearson’s r)	Gender (Spearman’s ρ)
First day of monitoring	-0.132	0.035
Days 2 to 10	-0.077	0.077
Days 11 to 20	-0.125	0.075
Days 21 to 30	-0.124	0.053

* p < 0.05, ** p < 0.01, *** p < 0.001.

**Fig. 3:**
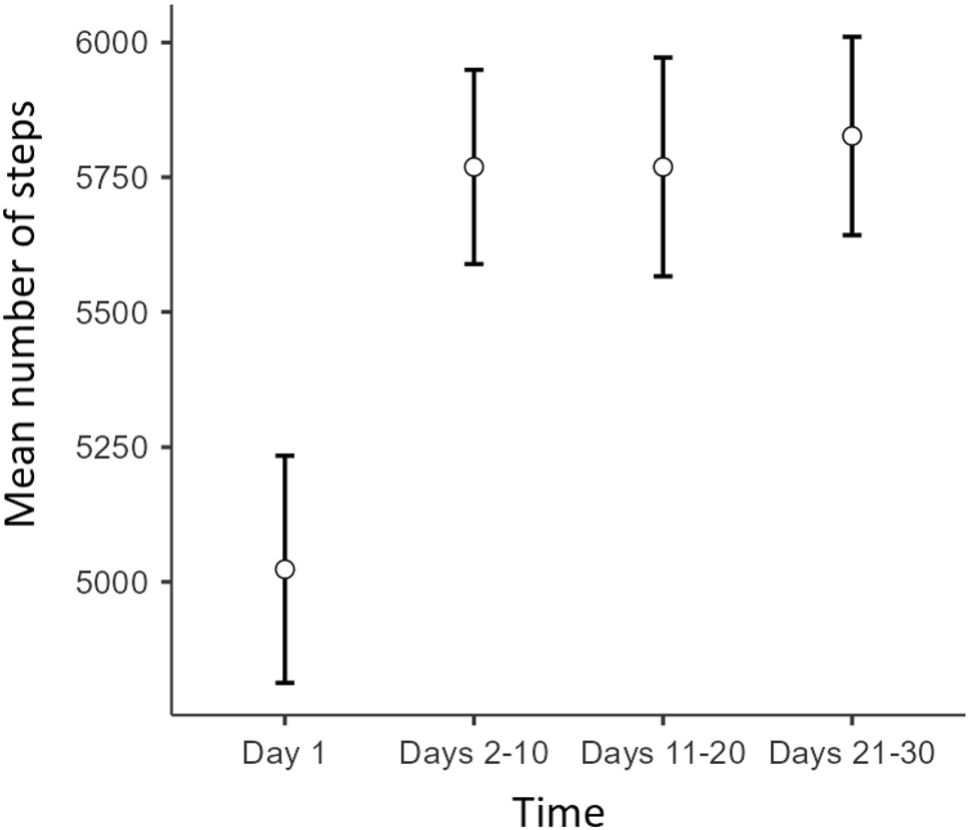
Participants’ mean number of steps over time. Error bars are standard errors.

A repeated measures ANOVA was performed to monitor the mean steps over the 30 days of intervention and directly compare the three experimental groups’ behavioral changes. The Mauchly sphericity test was significant (W = 0.856, p < 0.001), suggesting a violation of the sphericity assumption. Consequently, the Greenhouse–Geisser correction was used. The results revealed a significant effect of time (F = 8.18; p < 0.001; η^2^_p_ = 0.042; achieved power = 0.99). The mean number of daily steps increased from the first day (mean = 5023; SE = 211) to the following ten days of monitoring (mean = 5769; SE = 180) and remained stable over the following 10-days’ time-interval (mean = 5769; SE = 203) and the final 10-days’ time-interval (mean = 5827; SE = 184), as illustrated in Fig. 3. Bonferroni’s post-hoc tests suggested a significant difference between the mean steps of the first day and the following three-time intervals (p < 0.001). The other contrasts were not statistically significant.

**Fig. 4:**
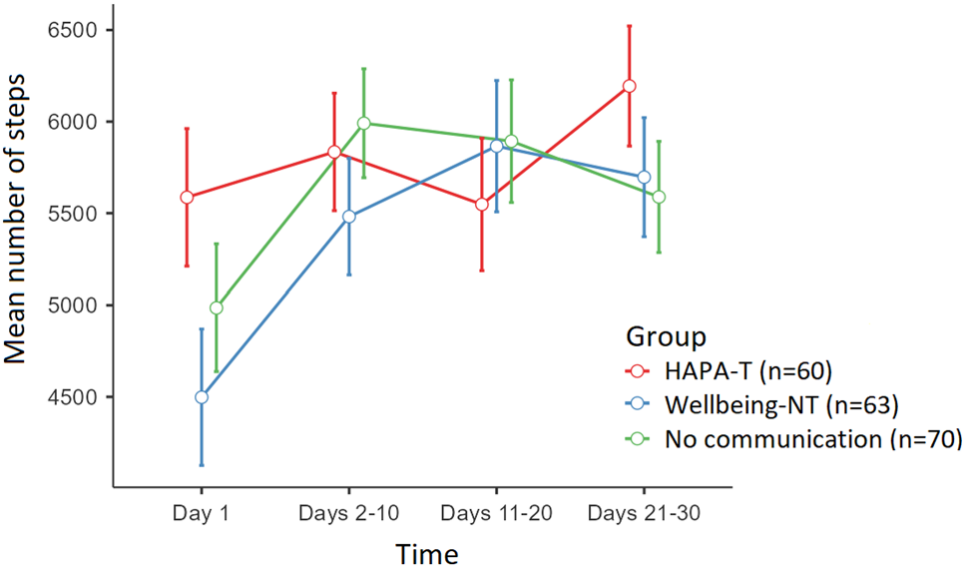
Mean number of steps of the three experimental groups over time.

The descriptive statistics suggested differences in the longitudinal trend of the mean step number depending on the experimental group. In particular, the HAPA-T group increased the mean step number at the final time point. The other two groups showed a rapid increase in the mean number of steps, which, however, tended to decrease at the final time-point (Fig. 4). However, this observation was not supported by statistical significance; indeed, the time x group interaction was not statistically significant (p = 0.063).

### Social-cognitive (HAPA) correlates of walking behavior change

Table 3 shows the correlation coefficients between the HAPA model variables and the walking behavior over time. Overall, the HAPA model variables were significantly related to each other. Regarding the motivational phase, significant positive correlations between action self-efficacy, intention to change, and walking behavior over time were estimated. Regarding the volitional phase, planning and maintenance self-efficacy were significantly correlated to the walking behavior observed in the last ten days of the trial.

**Table 3:** Correlation matrix (Pearson’s r) including the HAPA model variables and walking behavior.

	1	2	3	4	5	6	7	8	9	10
*Motivational phase*										
1. Action Self-efficacy	-									
2. Outcome Expectancies (positive)	.232**	-								
3. Outcome Expectancies (negative)	-.325***	.156*	-							
4. Risk Perception	-.229**	.033	.169*	-						
5. Intention to change	.344***	.186**	-.179*	-.034	-					
*Volitional phase*										
6. Planning	.324***	.184*	-.129	-.190**	.204**	-				
7. Maintenance Self-efficacy	.257***	.205**	-.182*	-.064	.297***	.333***	-			
8. Recovery Self-efficacy	.330***	.209**	-.137	-.200**	.216**	.447***	.434***	-		
9. Walking behavior (first day of monitoring)	.216**	.008	-.122	-.096	.145*	.109	.174*	.048	-	
10. Walking behavior (days 21 to 30)	.237***	.021	-.130	-.078	.237***	.208**	.181*	.060	.474***	-

* p < 0.05, ** p < 0.01, *** p < 0.001. Achieved power for r ≥ 0.3 was 0.99**Figures Legend.**

## Discussion

Advancements in theory-driven behavior change techniques^33,34^ and interventions focusing on health-related behavioral outcomes^12,31,32,36^ are essential for developing effective strategies to support primary prevention programs. Physical inactivity is a significant risk factor that researchers and clinicians have been targeting through innovative approaches, including tailored health communication^21–23^. This approach has increasingly integrated digital components^30^. Trials focusing on behavior change through mHealth have been widely conducted, showing promising but preliminary evidence regarding the effectiveness of digital tools^13–15^. The present study falls in this framework and is part of a broader research line that our research group has pursued, beginning with developing a theory-based trial protocol to assess the effectiveness of tailored print messages in promoting healthier lifestyles for individuals at risk of cardiovascular issues^25^. The current study aims to expand on the initial protocol by incorporating mobile tools, thus enhancing the reach of effective behavior change interventions.

A prospective, three-arm, parallel group, randomized study design was used to assess the impact of two types of communication interventions. The first intervention involved tailored communication using the Health Action Process Approach, as described previously^25^, while the second intervention focused on non-tailored communication related to wellbeing^35,38^. All participants were asked to achieve 7,000 steps daily and monitor their behavior, regardless of their assigned experimental group. This approach was based on evidence suggesting that persuasive communication is more effective when combined with specific goal-setting and self-monitoring strategies^12,31,32^.

The results revealed a significant and moderate main effect of time, indicating an overall improvement in walking behavior across all participants throughout the trial period. Specifically, it was found that the mean number of daily steps increased notably during the first ten days of the intervention and then remained statistically stable for the rest of the trial. Post-hoc analyses also showed significant differences in the mean steps between the initial monitoring day and the subsequent time intervals of the study. This finding aligns with previous mHealth interventions on physical activity, which have reported positive outcomes with small-to-moderate effect sizes^30^. Additionally, it is consistent with other research supporting the use of multiple behavior change techniques (BCTs)^31,32,36^. The increased walking observed in the group that did not receive any communication is also in line with research indicating that self-monitoring is a crucial technique for behavior change^12,34^.

The intervention had a significant overall impact. This evidence encourages using the overall intervention designed with the app in new contexts and with larger samples of individuals. However, it is not possible to draw conclusions about the differential effect of the type of communication. This result is different from previous findings suggesting that providing more tailored information increased the likelihood of positive behavior changes^21–23^.

The absence of a differential effects could be because all three experimental conditions contemplated multiple behavioral change techniques. Therefore, all three conditions resulted in increased physical activity, and the protocol used was not sufficiently sensitive to statistically detect a significant difference. Despite our efforts to have a large number of participants in each experimental group, it may be necessary to increase statistical power to detect statistically significant small changes in walking behavior.

Despite not being statistically significant, the mean daily step counts at the baseline were slightly different among the three study groups. This difference could be a confounding factor that affected the observed patterns over time.

However, when examining the data within each group, it was observed that participants exposed to tailored communication using the Health Action Process Approach displayed a more positive long-term trend than the others. Conversely, the remaining participants showed a trend in the opposite direction. Although this observation is qualitative, it aligns with the previous study by the research group^24^. As outlined in the introduction, the study aimed to assess the impact of a tailored print message intervention on increasing physical activity levels in patients with arterial hypertension. Those who received tailored printed material demonstrated a gradual increase in physical activity over twelve months. In contrast, patients who received non-tailored printed material initially increased their physical activity levels after six months of intervention, followed by a decrease. The similarity in trends observed in the present study is promising. It suggests the potential for expanding the study with a more extensive and well-balanced baseline physical activity sample, more extended intervention periods, and additional follow-up assessments to gain a deeper understanding of the effects of tailoring on physical activity trends.

According to the secondary aim of the study, a more in-depth investigation of the social-cognitive factors presumably underlying the longitudinal trajectories observed was then conducted. Correlational analyses were performed by linking the variables belonging to both the motivational and volitional phases of the HAPA with the walking behavior observed on the first day of monitoring and at the final stage of the intervention. Interestingly, significant correlations between action self-efficacy and all the study variables, including walking behavior, were estimated. This result finds support from prior studies on physical activity change that showed action self-efficacy to better explain intentions and behavior compared with studies on other health-related conducts^29^. Furthermore, maintenance self-efficacy and planning were significantly correlated to the walking behavior observed on the last days of the trial. Again, this finding corroborates what emerged from prior works evidencing the role of these volitional factors in promoting positive changes in physical activity^47,48^. However, it must be noted that these results should be taken as preliminary despite being informative. The associations observed should be investigated by considering the sequential structure of the HAPA model and, thus, by adopting a more longitudinal approach. In the present pilot study, the HAPA variables were all measured cross-sectionally, making it impossible to prospectively explore the social-cognitive determinants of walking behavior change. Future mHealth intervention studies may benefit from the present preliminary data and attempt to deepen the longitudinal effects of motivational and volitional factors through more complex analytical approaches (e.g., structural equation modeling).

Some limitations should be acknowledged. First, no process evaluations were performed at this pilot stage, which makes it difficult to generalize the present findings. Process evaluation involves using various measures to assess fidelity (i.e., whether the intervention was delivered as planned) and implementation quality, clarify causal mechanisms, and identify contextual factors associated with outcome variations. According to process evaluation guidelines^49,50^, an intervention may have limited effects either due to design weakness or to non-optimal implementation. Similarly, positive outcomes can be observed even when an intervention was not delivered fully as intended. To overcome this limit, iterative checks (e.g., momentary assessments, ad-hoc items) throughout the trial can unveil, for example, intervention fidelity and reach (i.e., whether the intervention was delivered and the participants were exposed to intervention contents as intended). Concretely, next studies may further test the implementation of the present intervention by systematically replicate monitoring measures throughout the study flow, ultimately identifying possible causal mechanisms and contextual factors that are related to the variation in the outcomes and, thus, ensuring stronger evidence robustness and replicability^49,50^. Secondly, it should be noted that a self-report methodology was adopted. While this approach may suffer from potential bias (e.g., risk of false-positive, social desirability), its deployment for the evaluation of health behaviors is widespread, as it relies on high practicality, satisfactory cost-effectiveness, and ability to capture subjective experience^51^. It is important to note that the measurement of daily steps provided a more objective indicator of walking behavior than self-report questionnaires. This method represents an initial step toward a more standardized and real-time evaluation of the primary outcome. However, in this study, the standardization was limited since participants may or may not own a smartwatch. The step data reported by smartphones and wearable devices, such as smartwatches, may not align perfectly. Future studies could address this limitation by supplying participants with wearable devices. A final limitation is that the psychological constructs of the HAPA were only measured at baseline. Future studies could test the effectiveness of theory-based interventions by evaluating whether the experimental manipulation impacts both psychological constructs and behavior.

In conclusion, some of the study’s strengths should also be recognized. First, the present study was specifically based on a theoretical model that has been widely implemented to investigate health promotion^29^. This model was adopted along with a tailored health communication approach, whose combination represents the novelty of the present contribution. A second strength is related to the digital approach adopted. It not only allowed us to test the preliminary efficacy of an intervention based on the use of an innovative strategy, but it also contributed to scaling up theory-based techniques focused on health behavior change. As previously mentioned, the increasing dissemination and the added value of mobile technology interventions have been discussed as a possible solution for overcoming scalability challenges^11,12^, but the lack of clear evidence of the effectiveness of mHealth programs was also recognized^13–15^. This underlined the need for further investigation that can empirically confirm the potential of digital tools in providing optimized and more transferable health behavior change interventions.

## Supplementary Information

Supplementary Information.

## Data Availability

The datasets generated and/or analysed during the current study are not publicly available due to privacy or ethical restrictions but are available from the corresponding author on reasonable request.

## References

[1] Non communicable diseases. https://www.who.int/news-room/fact-sheets/detail/noncommunicable-diseases (2024).

[2] Timmis, A. et al. European society of cardiology: Cardiovascular disease statistics 2021. *Eur. Heart J.* **43**, 716–799 (2022). 35016208 10.1093/eurheartj/ehab892

[3] Physical activity. https://www.who.int/news-room/fact-sheets/detail/physical-activity (2024).

[4] Duran, A. T. et al. Breaking up prolonged sitting to improve cardiometabolic risk: Dose-response analysis of a randomized crossover trial. *Med. Sci. Sports Exerc.* **55**, 847–855 (2023). 36728338 10.1249/MSS.0000000000003109

[5] Pinto, A. J. et al. Physiology of sedentary behavior. *Physiol. Rev.* **103**, 2561–2622 (2023). 37326297 10.1152/physrev.00022.2022 PMC10625842

[6] Garber, C. E. et al. American College of Sports Medicine position stand. Quantity and quality of exercise for developing and maintaining cardiorespiratory, musculoskeletal, and neuromotor fitness in apparently healthy adults: guidance for prescribing exercise. *Med. Sci. Sports Exerc.* **43**, 1334–1359 (2011). 21694556 10.1249/MSS.0b013e318213fefb

[7] Paluch, A. E. et al. Steps per day and all-cause mortality in middle-aged adults in the coronary artery risk development in young adults study. *JAMA Netw. Open* **4**, e2124516 (2021). 34477847 10.1001/jamanetworkopen.2021.24516 PMC8417757

[8] Tudor-Locke, C. & Bassett, D. R. How many steps/day are enough? Preliminary pedometer indices for public health. *Sports Med. Auckl. NZ* **34**, 1–8 (2004). 10.2165/00007256-200434010-00001 14715035

[9] The Global Status Report on Physical Activity 2022. https://www.who.int/teams/health-promotion/physical-activity/global-status-report-on-physical-activity-2022.

[10] World Health Organization. *Global Action Plan on Physical Activity 2018–2030: More Active People for a Healthier World* (World Health Organization, 2018).

[11] Hosseinpour, M. & Terlutter, R. Your personal motivator is with you: A systematic review of mobile phone applications aiming at increasing physical activity. *Sports Med. Auckl. NZ* **49**, 1425–1447 (2019). 10.1007/s40279-019-01128-3 PMC6684571 31144235

[12] Schroé, H. et al. Which behaviour change techniques are effective to promote physical activity and reduce sedentary behaviour in adults: A factorial randomized trial of an e- and m-health intervention. *Int. J. Behav. Nutr. Phys. Act.* **17**, 127 (2020). 33028335 10.1186/s12966-020-01001-x PMC7539442

[13] Chaudhry, U. A. R. et al. The effects of step-count monitoring interventions on physical activity: Systematic review and meta-analysis of community-based randomised controlled trials in adults. *Int. J. Behav. Nutr. Phys. Act.* **17**, 129 (2020). 33036635 10.1186/s12966-020-01020-8 PMC7545847

[14] Direito, A., Carraça, E., Rawstorn, J., Whittaker, R. & Maddison, R. mHealth technologies to influence physical activity and sedentary behaviors: Behavior change techniques, systematic review and meta-analysis of randomized controlled trials. *Ann. Behav. Med.* **51**, 226–239 (2017). 27757789 10.1007/s12160-016-9846-0

[15] Romeo, A. et al. Can smartphone apps increase physical activity? Systematic review and meta-analysis. *J. Med. Internet Res.* **21**, e12053 (2019). 30888321 10.2196/12053 PMC6444212

[16] D’Addario, M., Baretta, D., Zanatta, F., Greco, A. & Steca, P. Engagement features in physical activity smartphone apps: Focus group study with sedentary people. *JMIR MHealth UHealth* **8**, e20460 (2020). 33196450 10.2196/20460 PMC7704278

[17] Hoj, T. H. et al. How do apps work? An analysis of physical activity app users’ perceptions of behavior change mechanisms. *JMIR MHealth UHealth* **5**, e114 (2017). 28778846 10.2196/mhealth.7206 PMC5561388

[18] Kowatsch, T., Otto, L., Harperink, S., Cotti, A. & Schlieter, H. A design and evaluation framework for digital health interventions. *It - Inf. Technol.* **61**, 253–263 (2019).

[19] Reis, R. S. et al. Scaling up physical activity interventions worldwide: stepping up to larger and smarter approaches to get people moving. *The Lancet* **388**, 1337–1348 (2016). 10.1016/S0140-6736(16)30728-0 PMC5193005 27475273

[20] Short, C. E. et al. Measuring engagement in ehealth and mhealth behavior change interventions: Viewpoint of methodologies. *J. Med. Internet Res.* **20**, e292 (2018). 30446482 10.2196/jmir.9397 PMC6269627

[21] Kreuter, M. W., Strecher, V. J. & Glassman, B. One size does not fit all: The case for tailoring print materials. *Ann. Behav. Med.* **21**, 276–283 (1999). 10721433 10.1007/BF02895958

[22] Noar, S. M., Benac, C. N. & Harris, M. S. Does tailoring matter? Meta-analytic review of tailored print health behavior change interventions. *Psychol. Bull.* **133**, 673–693 (2007). 17592961 10.1037/0033-2909.133.4.673

[23] Pope, J. P., Pelletier, L. & Guertin, C. Starting off on the best foot: A review of message framing and message tailoring, and recommendations for the comprehensive messaging strategy for sustained behavior change. *Health Commun.* **33**, 1068–1077 (2018). 28622007 10.1080/10410236.2017.1331305

[24] Adorni, R. et al. Effectiveness of a tailored communication intervention to improve physical activity in hypertensive patients: A twelve-month randomized controlled trial. *BMC Cardiovasc. Disord.* **24**, 143 (2024). 38443805 10.1186/s12872-024-03786-2 PMC10913652

[25] D’Addario, M., Cappelletti, E. R., Sarini, M., Greco, A. & Steca, P. The TTCYB study protocol: A tailored print message intervention to improve cardiovascular patients’ lifestyles. *Int. J. Environ. Res. Public. Health* **17**, 2919 (2020). 32340219 10.3390/ijerph17082919 PMC7215990

[26] Schwarzer, R. Modeling health behavior change: How to predict and modify the adoption and maintenance of health behaviors. *Appl. Psychol.* **57**, 1–29 (2008).

[27] Hardcastle, S. J., Maxwell-Smith, C. & Hagger, M. S. Predicting physical activity change in cancer survivors: An application of the Health Action process approach. *J. Cancer Surviv.* **16**, 1176–1183 (2022). 34518960 10.1007/s11764-021-01107-6 PMC9630182

[28] Steca, P. et al. Changes in physical activity among coronary and hypertensive patients: A longitudinal study using the Health Action Process Approach. *Psychol. Health* **32**, 361–380 (2017). 28049344 10.1080/08870446.2016.1273353

[29] Zhang, C.-Q., Zhang, R., Schwarzer, R. & Hagger, M. S. A meta-analysis of the health action process approach. *Health Psychol.* **38**, 623–637 (2019). 30973747 10.1037/hea0000728

[30] Davis, A., Sweigart, R. & Ellis, R. A systematic review of tailored mHealth interventions for physical activity promotion among adults. *Transl. Behav. Med.* **10**, 1221–1232 (2020). 33044542 10.1093/tbm/ibz190

[31] Carfora, V., Catellani, P., Caso, D. & Conner, M. How to reduce red and processed meat consumption by daily text messages targeting environment or health benefits. *J. Environ. Psychol.* **65**, 101319 (2019).

[32] Michie, S. et al. A refined taxonomy of behaviour change techniques to help people change their physical activity and healthy eating behaviours: The CALO-RE taxonomy. *Psychol. Health* **26**, 1479–1498 (2011). 21678185 10.1080/08870446.2010.540664

[33] Chase, J.-A.D., Otmanowski, J., Rowland, S. & Cooper, P. S. A systematic review and meta-analysis of interventions to reduce sedentary behavior among older adults. *Transl. Behav. Med.* **10**, 1078–1085 (2020). 33044538 10.1093/tbm/ibz189

[34] Michie, S., Abraham, C., Whittington, C., McAteer, J. & Gupta, S. Effective techniques in healthy eating and physical activity interventions: a meta-regression. *Health Psychol. Off. J. Div. Health Psychol. Am. Psychol. Assoc.* **28**, 690–701 (2009). 10.1037/a0016136 19916637

[35] Caso, D., Carfora, V., Capasso, M., Oliano, D. & Conner, M. Using messages targeting psychological versus physical health benefits to promote walking behaviour: A randomised controlled trial. *Appl. Psychol. Health Well-Being* **13**, 152–173 (2021). 32945103 10.1111/aphw.12224

[36] Patterson, K. et al. Behaviour change techniques in cardiovascular disease smartphone apps to improve physical activity and sedentary behaviour: Systematic review and meta-regression. *Int. J. Behav. Nutr. Phys. Act.* **19**, 81 (2022). 35799263 10.1186/s12966-022-01319-8 PMC9261070

[37] Bell, L. et al. How notifications affect engagement with a behavior change app: Results from a micro-randomized trial. *JMIR MHealth UHealth* **11**, e38342 (2023). 37294612 10.2196/38342 PMC10337295

[38] MacPherson, M. M., Cranston, K. D., Locke, S. R., Bourne, J. E. & Jung, M. E. Using the behavior change wheel to develop text messages to promote diet and physical activity adherence following a diabetes prevention program. *Transl. Behav. Med.* **11**, 1585–1595 (2021). 34008852 10.1093/tbm/ibab058 PMC8604265

[39] Cohen, J. *Statistical Power Analysis for the Behavioral Sciences* (Academic Press, 2013).

[40] Faul, F., Erdfelder, E., Lang, A.-G. & Buchner, A. G*Power 3: A flexible statistical power analysis program for the social, behavioral, and biomedical sciences. *Behav. Res. Methods* **39**, 175–191 (2007). 17695343 10.3758/bf03193146

[41] Craig, C. L. et al. International physical activity questionnaire: 12-country reliability and validity. *Med. Sci. Sports Exerc.* **35**, 1381–1395 (2003). 12900694 10.1249/01.MSS.0000078924.61453.FB

[42] Mannocci, A. *et al.* International Physical Activity Questionnaire: validation and assessment in an Italian sample. *Ital. J. Public Health* **7**, (2010).

[43] West, S. G., Finch, J. F. & Curran, P. J. Structural equation models with nonnormal variables: Problems and remedies. in *Structural equation modeling: Concepts, issues, and applications* 56–75 (Sage Publications, Inc, Thousand Oaks, CA, US, 1995).

[44] Hayes, A. F. & Coutts, J. J. Use Omega Rather than Cronbach’s Alpha for Estimating Reliability. *Commun. Methods Meas.* **14**, 1–24 (2020).

[45] McDonald, R. P. *Test Theory: A Unified Treatment* (Psychology Press, 2013).

[46] Hansen, W. B., Tobler, N. S. & Graham, J. W. Attrition in substance abuse prevention research: A meta-analysis of 85 longitudinally followed cohorts. *Eval. Rev.* **14**, 677–685 (1990).

[47] Caudroit, J., Boiché, J. & Stephan, Y. The role of action and coping planning in the relationship between intention and physical activity: A moderated mediation analysis. *Psychol. Health* **29**, 768–780 (2014). 24446685 10.1080/08870446.2014.884223

[48] Di Maio, S., Keller, J., Hohl, D. H., Schwarzer, R. & Knoll, N. Habits and self-efficacy moderate the effects of intentions and planning on physical activity. *Br. J. Health Psychol.* **26**, 50–66 (2021). 32584510 10.1111/bjhp.12452

[49] Steckler, A. & Linnan, L. *Process Evaluation for Public Health Interventions and Research*. Jossey-Bass/Wiley (2002).

[50] Moore, G. et al. Process evaluation in complex public health intervention studies: The need for guidance. *J. Epidemiol. Community Health* **68**, 101–102 (2014). 24022816 10.1136/jech-2013-202869 PMC3892708

[51] Prince, S. A. et al. A comparison of direct versus self-report measures for assessing physical activity in adults: A systematic review. *Int. J. Behav. Nutr. Phys. Act.* **5**, 56 (2008). 18990237 10.1186/1479-5868-5-56 PMC2588639

