# Supplementary material for: Efficacy of a theory-based and tailored mHealth intervention promoting walking behavior: a preliminary randomized controlled trial
Source: Sci Rep. 2025 Jul 18;15:26033. doi: 10.1038/s41598-025-09634-3 (PMC12271361; doi:10.1038/s41598-025-09634-3)
Supplement: Supplementary file 1 — Supplementary Information. [file 41598_2025_9634_MOESM1_ESM.pdf]

Supplementary Material

Table S1. Tailored scheme for the development of notifications aimed at achieving 7,000 steps per day (HAPA-T group)

**Self-report Questionnaire (Baseline)**<br>**Social-Cognitive variable**	**Scoring**	**Intervention**<br>**Example notification**
Action Self-Efficacy<br>"How capable do you feel of walking regularly?"	Low	*You do not feel very capable of reaching the 7,000 steps goal, but with good planning and the right strategies, you can walk regularly without much effort!*
	High	*You feel very capable of reaching the goal of 7,000 steps: it is an excellent starting point for achieving and maintaining regular physical activity over time!*
Health risk perception<br>"Considering how much I walk, I think I am personally at risk for heart disease."	Low	*You need to be clearer on the effect of physical activity on the development of cardiovascular diseases: regular walking will help your heart feel good.*
	High	*You know well that regular physical activity protects against the development of cardiovascular diseases, so increase the number of steps you take daily to stay healthy.*
Positive outcome expectancies<br>"If I walk at least 7,000 steps daily, I expect to meet new people."	Low	*You underestimate how much regular physical activity can allow you to meet new people: take this opportunity to reach your 7,000 daily steps!*
	High	*Walking regularly outdoors allows you to meet new people: one more reason to increase your daily step count!*
Negative outcome expectancies<br>"If I walk at least 7,000 steps daily, I will get exhausted."	Low	*You rightly do not expect to tire by walking regularly: use this awareness to improve your physical activity!*
	High	*It can certainly be challenging to start walking regularly, but you will see that it will help you feel fitter over time!*
Planning<br>"In taking at least 7,000 steps a day, I have a detailed plan for where I can walk during the day."	Low	*You have a few ideas about WHERE you can walk: find, for example, a nearby park or opt for the quieter streets of your neighborhood.*
	High	*You know well which places and itineraries will allow you to achieve your daily goals: it is an excellent starting point!*
Maintenance self-efficacy<br>"I will be able to continue walking at least 7,000 steps a day even when I feel depressed."	Low	*If you think you cannot walk regularly when your spirits are low, do not give up because physical activity is also good for your mood!*
	High	*Even when you are in a bad mood, you think you can still maintain regular physical activity: use this to your advantage to achieve your goal!*
Recovery self-efficacy<br>"Despite good intentions, mistakes and relapses can happen. How capable will you feel of starting to change your habits again?"	Low	*You may not reach your goal of 7,000 steps, but be careful not to be discouraged: always work to improve your health!*
	High	*It may happen that you do not reach the goal of 7,000 steps, but this thought does not discourage you: use this belief to maintain regular physical activity.*

Table S2. Text of the notifications used for the non-tailored communication focusing on wellbeing (Wellbeing-NT group)

Day	Notification
1	*The more you walk regularly, the more you strengthen your emotional wellbeing.*
2	*Walking regularly every day helps you reduce bad mood.*
3	*The more you walk regularly, the more you stimulate the production of the "happiness hormone" (serotonin).*
4	*Walk more and decrease the nervousness caused by stress.*
5	*Walking regularly outside improves your mood.*
6	*Walking regularly every day helps you reduce the feeling of mental slowdown!*
7	*Walking in the morning will wake you up and give you more mental energy throughout the day.*
8	*The more you walk regularly, the more you improve your self-esteem.*
9	*The number of steps you take and your general mood are strongly related*
10	*Walking regularly helps you fight negative emotions, such as guilt for not being physically active.*
11	*Walking outside frees the mind.*
12	*Walk every day and avoid apathy.*
13	*The more you walk regularly, the more you keep your mind active.*
14	*Walking regularly is associated with lower depression risk.*
15	*The number of steps you take each day improves many psychological aspects.*
16	*The more you walk every day, the more you reduce the irritability caused by stress.*
17	*Walking regularly triggers the creation of dopamine, which helps you aspire to achieve goals and take action to reach them.*
18	*The more you walk regularly, the more you have relief from unpleasant emotions.*
19	*Walk every day and improve your self-esteem.*
20	*The more steps you take, the more you reduce anxiety.*
21	*In the morning, you can benefit from a boost of mental energy from a walk.*
22	*Walking regularly will help you feel emotionally less tense.*
23	*The more steps you take in a day, the more you feel you have mental concentration.*
24	*Humans are not machines. You need to move away from daily emotional stressors. Do it by walking!*
25	*The more steps you take, the more you increase your creativity.*
26	*Walking regularly reduces negative feelings associated with physical inactivity.*
27	*Walking at the end of the day helps you relax and refresh your ideas.*
28	*The more you walk, the more you can reduce negative emotions.*
29	*Walk more and feel more peaceful.*
30	*Walking regularly every day helps prevent sleepiness.*
